# *Astragalus membranaceus* Injection Suppresses Production of Interleukin-6 by Activating Autophagy through the AMPK-mTOR Pathway in Lipopolysaccharide-Stimulated Macrophages

**DOI:** 10.1155/2020/1364147

**Published:** 2020-07-04

**Authors:** Xiaoyan Zhang, Taigang Liang, Wanxia Yang, Lanfang Zhang, Shuting Wu, Chaoqun Yan, Qingshan Li

**Affiliations:** ^1^School of Pharmaceutical Science, Shanxi Medical University, Taiyuan 030001, China; ^2^Shanxi Key Laboratory of Innovative Drug for the Treatment of Serious Diseases Basing on the Chronic Inflammation, Shanxi University of Chinese Medicine, Jinzhong 030619, China; ^3^Department of Laboratory Medicine, Fenyang College of Shanxi Medical University, Fenyang 032200, China

## Abstract

*Astragalus membranaceus* (AM), used in traditional Chinese medicine, has been shown to enhance immune functions, and recently, its anti-inflammatory effects were identified. However, the mechanisms of action remain unclear. Most studies have shown that autophagy might be involved in the immune response of the body, including inflammation. Here, we developed an inflammatory model by stimulating macrophages with lipopolysaccharides (LPS) to explore the anti-inflammatory effect and mechanisms of AM injection from the perspective of the regulation of autophagy. Immunoblot, immunofluorescence, and ELISA were used to determine the effects of AM injection on the production of interleukin-6 (IL-6) and alterations of autophagy markers. It was found that AM injection reduced the expression of IL-6 in LPS-stimulated macrophages and reversed the LPS-induced inhibition of cellular autophagy. After treatment with inhibitors of signaling pathways, it was shown that LPS downregulated autophagy and upregulated the production of IL-6 in macrophages via the protein kinase B (Akt)/mammalian target of rapamycin (mTOR) pathway. AM injection reversed the effects of LPS by activating the AMP-activated protein kinase (AMPK) instead of inhibiting Akt. These results were further confirmed by testing activators and siRNA silencing of AMPK. Hence, these 2 distinct signaling molecules appear to exert opposite effects on mTOR, which integrates information from multiple upstream signaling pathways, negatively regulating autophagy. In addition, we demonstrated that autophagy might play a key role in regulating the production of IL-6 by testing activators and inhibitors of autophagy and siRNA silencing of *ATG5*. These findings showed that AM injection might enhance autophagy by activating AMPK and might further play a repressive effect on the LPS-stimulated expression of IL-6. This study explored the relationship between autophagy, signaling pathways, and the production of inflammatory factors in a model of endotoxin infection and treatment with AM injection.

## 1. Introduction

Recent studies have linked inflammation to the development of a number of important diseases [1–4]. Inflammation can be induced by various stimuli, such as damage, pathogens, and their products, and is often accompanied by immune disorders [5–7]. Accordingly, it has been shown that anti-inflammatory treatment alone might not be usually effective [8]. Moreover, long-term application of anti-inflammatory drugs might also cause side effects [9, 10], such as adverse cardiovascular profiles, renal dysfunction, and blood pressure elevation as in the case of administration of nonsteroidal anti-inflammatory drugs [11]. Therefore, it is necessary to develop novel anti-inflammatory drugs with high security, which could also protect the body from the pathological damage induced by inflammation. Some original and review papers have reported the anti-inflammatory effects of medicinal plants and isolated natural products and proposed their use in potential anti-inflammatory treatments [7, 12]. Lipopolysaccharides (LPS) are known as a common pathogen-associated molecular pattern (PAMP) and inflammatory inducer. There have been many experimental evidence suggesting that various inflammatory mediators or markers, such as tumor necrosis factor-*α* (TNF-*α*), interleukin-1*β* (IL-1*β*), and interleukin-6 (IL-6), are substantially elevated in LPS-stimulated macrophagocytes [13–15]. IL-6, a central proinflammatory cytokine that is considered a key hallmark of inflammation in the body, is known to be promptly produced in response to infectious factors and tissue injuries and can regulate intracellular signal transduction and affect other cytokines [16–19]. Most importantly, the acute expression of IL-6 has been reported to be a critical driving factor for many diseases [20, 21]. Accordingly, the regulation of the expression of IL-6 could be used as an entry point for the treatment of inflammation and other diseases caused by inflammation [19, 22, 23]. Therefore, in this study, we investigated the production of IL-6 to estimate the inflammatory response of LPS-stimulated macrophages.

*Astragalus membranaceus* (AM) is a traditional Chinese medicine widely used in clinical therapy and health care. It has been reported to exert a wide range of biological activities, such as enhanced immune functions [24], strengthened cardiac functions [25], antidiabetic properties [26], and antitumor [27], antiviral [28], antioxidant [29, 30], and longevity effects [31]. In recent years, several studies have found that AM exhibits also anti-inflammatory effects by regulating the secretion of inflammatory factors [32–34]. Adesso et al. [33] showed that the extract of AM reduced the release of tumor necrosis factor-*α* (TNF-*α*), and the expression of cycloxygenase-2 (COX-2) and inducible nitric oxide synthase (iNOS), the activation of nuclear factor-*κ*B (NF-*κ*B), and the release of reactive oxygen species (ROS) in a nontumorigenic intestinal epithelial cell line (IEC-6) induced by LPS plus interferon-*γ* (IFN). Guo et al. [35] found that *Astragalus* polysaccharides could reduce the mRNA expression of the inflammatory IL-6 cytokine both in vivo and in vitro. However, the anti-inflammatory mechanism of AM has not been investigated entirely. In view of the fact that it has been shown to exert both enhanced physical function and anti-inflammatory effects [36], as well as its long-term safety, AM might therefore be suitable for utilization in the clinical treatment of inflammation and other diseases caused by inflammation. An AM injection constitutes a type of standard extract of AM with clear composition and stable quality, strictly prepared and identified according to the standard of the Chinese Pharmacopoeia of the Ministry of Health of the People's Republic of China [37]. Although the AM injection has shown some therapeutic effects in clinical treatment settings [38–41], its application in anti-inflammatory treatments has been rare.

Autophagy is a highly conserved cellular process that eliminates damaged organelles or defective proteins to facilitate cell survival and adaptation, while maintaining homeostasis during starvation, genotoxic stress, and oxidative stress in normal cells [42]. Autophagy is generally considered to have beneficial effects on health and lifespan [43, 44]. Moreover, defective autophagy has been linked to several pathological conditions, such as infections, inflammation, and tumors [45, 46]. Recently, increasing studies have demonstrated that autophagy could inhibit the overproduction of inflammatory cytokines, thereby alleviating cellular injury [47–49]. In contrast, Ding et al. [50] reported that the autophagy inhibitor 3-methyladenine (3-MA) could reverse an LPS-induced lung injury through the inhibition of autophagy and inflammation, indicating that autophagy was involved in inflammation. Collectively, these observations have shown the protective or detrimental effect of autophagy in inflammation. However, further work is required to uncover the role of autophagy and its associated mechanisms in inflammation. It has also been reported that LPS could induce autophagy in bone marrow-derived macrophages [51]. A recent study, however, showed that LPS inhibited autophagy and caused pulmonary microvascular barrier damage, with autophagy regulating the therapeutic potential of adipose-derived stem cells in a LPS-induced model [52]. Hence, both the influence of LPS on autophagy and the regulatory effect of autophagy on inflammation remain unclear and need further exploration.

Autophagy is under the control of multiple signaling events converging on a single mediator, the kinase mTOR, a major suppressor of the initiation of autophagy. The roles of these pathways in the formation of phagophores and initiation of autophagy are relatively well understood. The phosphatidylinositol-3-kinase (PI3K)/Akt signaling pathway has been shown to inhibit autophagy by activating mTOR. The 5′ AMP-activated protein kinase (AMPK) is a multimeric serine/threonine protein kinase. Both, adenosine monophosphate (AMP) and adenosine diphosphate (ADP) are known to promote the activity of AMPK, which thus acts as a sensor of the cellular energy charge that maintains cellular energy homeostasis [53]. Activation of AMPK has been recognized to be also involved in the initiation of autophagy through the inhibition of the downstream mTOR kinase. Many active natural products in vegetables, fruits, and medicinal and edible plants have been found to regulate autophagy [54]. Various active ingredients, such as quercetin [55], resveratrol [56], and curcumin [57], were demonstrated to regulate autophagy through mTOR. Accordingly, extracts of AM have also been found to exert regulatory effects on autophagy-related diseases, such as antioxidation [58], anti-inflammation [57], anticancer [59], and skin antiaging [42] effects [60]. As such, the mechanisms of AM affecting autophagy have begun to be revealed.

In this study, we evaluated the expression of IL-6 to verify whether AM injection could play an anti-inflammatory role in LPS-stimulated macrophages and investigated the effects and mechanisms exerted by LPS on autophagy and whether autophagy was involved in regulating the production of the IL-6 induced by LPS. Finally, we explored whether there was a crosstalk between the anti-inflammatory effect of AM and its regulatory effect on autophagy.

## 2. Materials and Methods

### 2.1. Materials

ANA cells were purchased from Shanghai Institute of Cell Biology, Chinese Academy of Sciences (Shanghai, China). Fetal bovine serum was purchased from CellMax Company (Beijing, China). Rapamycin, LY294002, and Torin 1 were purchased from Selleck Co. Ltd. (Shanghai, China), and a mouse IL-6 ELISA kit was purchased from BioLegend, Inc. (San Diego, CA, USA). BCA protein assay kit, ECL chemiluminescence kit, RPMI 1640 medium, antibiotic cocktail (mixture of penicillin and streptomycin), peroxidase-conjugated goat anti-mouse IgG antibody (#BA1050), and peroxidase-conjugated goat anti-rabbit IgG antibody (#BA1054) were purchased from Boster Biological Technology Co. Ltd. (Wuhan, China). X-ray Films (XBT 6535876), Fixer, and Replenisher were purchased from Carestream Health, Inc. 3-Methyladenine and SQSTM/p62 antibody (#P0067) were purchased from Sigma Co. LLC (Shanghai, China); phospho-AMPK alpha (Thr172) (#AF3423) and AMPK alpha (BF2001) antibodies were purchased from Affinity Biosciences; and *β*-actin antibody (AP0060) was purchased from Bioworld Technology, Inc. (Nanjing, China). IL-6 antibody (GB11117) was purchased from Wuhan Servicebio Technology Co. Ltd. (Wuhan, China). Cell lysis buffer, SDS-PAGE gel preparation kit, and FITC-labeled goat anti-rabbit IgG (A0562) and Cy3-labeled goat anti-rabbit IgG (A0516) antibodies were purchased from Beyotime Biotechnology Company. mTOR (#2983), phospho-mTOR (Ser2481) (#2974), phospho-Akt (Ser473) (#4060), Akt (#4685), and LC3 (#12741) antibodies were purchased from Cell Signaling Technology Inc. (Shanghai, China). AMPK siRNA was synthesized by GenePharma Company (Shanghai, China). *Astragalus membranaceus* injection was purchased from Zhengda Qingchunbao Pharmaceutical Co. Ltd. (Hangzhou, China); the effective composition of every 10 mL injection was equivalent to 20 g of raw herbs. The content of astragaloside IV (C41H68O14), used as the quality standard, was not less than 0.08 mg per mL. In this study, we converted the dose of AM injection into the original concentration of AM, and henceforth, they are referred to as “AM” uniformly.

### 2.2. Cell Culture and Experimental Group

Mouse macrophage ANA-1 cells were inoculated in RPMI 1640 medium containing 10% fetal bovine serum (FBS) and 1% antibiotic cocktail (mixture of penicillin and streptomycin) and cultured in an incubator containing 5% CO_2_ at 37°C. The medium was replaced every 18-24 h. Cells passage were performed when they reached 80 to 90% confluency. Accordingly, ANA-1 cells in the logarithmic growth phase were adjusted to 1 × 10^6^ cells/mL in RPMI 1640 complete medium and inoculated into 6-well plates in aliquots of 2 mL per well and cultured for 12 h. Cells from each group were exposed to LPS, AM injection, inhibitors, and activators and cultured for varying hours.

### 2.3. Western Blot Analysis

Cells were collected by centrifugation at 2500 rpm for 3 min. Total protein was extracted from cells using cell lysis buffer. Protein concentration was measured using a BCA protein assay kit, and protein was denatured by adding 5× loading buffer and heated at 95°C for 5 min. Approximately 30 *μ*g total protein from each group was separated by 12% SDS-PAGE and transferred to a nitrocellulose (NC) or polyvinylidene fluoride (PVDF) membrane. After blocking for 1-2 h at 25°C in 5% skim milk powder in TBS solution, membranes were incubated with LC3 (1 : 1000), p62 (1 : 3000), *β*-actin (1 : 1500), IL-6 (1 : 1000), AMPK (1 : 1000), p-AMPK (1 : 500), Beclin 1 (1 : 1000), ATG5 (1 : 1000), mTOR (1 : 1000), p-mTOR (1 : 1000), p-Akt (1 : 1000), and Akt (1 : 1000) antibodies at 4°C overnight. This was followed by incubation with appropriate secondary antibodies (1 : 5000) for 2 h at 25°C, and then, an ECL kit was used for detection. Gray value analysis was performed using the ImageJ software (NIH, Bethesda, MD, USA). Relative protein expression was given by the following equation: relative protein expression = gray value per sample strip/gray value of *β*‐actin per sample strip.

### 2.4. ELISA

The supernatant of the culture media from each group was collected and centrifuged at 2500 rpm for 3 min. Then, 100 *μ*L supernatant was added to enzyme plate wells coated with IL-6 antibodies. Three repeated wells were used for each sample. The plate was incubated at 25°C for 2 h. After washing the plate, the enzyme-labeled antibody was added, incubated at 25°C for 1 h, and following another washing, the substrate and colorant were added. This step was performed in the dark at 25°C for 20 min. Finally, the termination solution was added and absorbance values were read using a microplate spectrophotometer at 450 nm and 570 nm.

### 2.5. siRNA Transfection

ANA-1 cells were plated into 6-well plates (2.0 × 10^5^/well) and cultured for 18 h so that the degree of cell fusion reached 50% during transfection. Consecutively, 120 pmol siRNA was added to 200 *μ*L fresh medium (without bovine serum and antibiotics) and gently mixed. Then, 5 *μ*L of GP-siRNA-Mate transfection reagent was added to the above medium, gently mixed, and allowed to stand at 25°C for 10 min. The mixture was then added to the six-well plate which had been replaced with fresh complete media in advance. Cells were cultured for 48 h before treating.

### 2.6. Immunofluorescence Staining

Cells in the log phase were cultured in 24-well plates. After treatment, the plate was centrifuged and washed with phosphate buffer solution (PBS). Each group of cells was fixed with 4% paraformaldehyde PBS and then treated with 0.5% Triton X-100 PBS. Cells were blocked with 5% bovine serum albumin (BSA) in PBS for 30 min at 25°C, followed by an overnight incubation at 4°C with primary antibodies: LC3 (1 : 200) and p62 (1 : 1000). After washing with PBS, cells were incubated with Cy3 goat anti-rabbit IgG antibody or FITC goat anti-rabbit IgG antibody for 1 h at 25°C. Then, they were washed with PBS before nuclear staining with 4′,6-diamidino-2-phenylindole (DAPI) and observation under a fluorescent microscope (Nikon Corporation, Tokyo, Japan) and detection using high content imaging systems (Molecular Devices Corporation, San Jose, USA).

### 2.7. Statistical Analysis

Data were expressed as mean ± standard deviation (SD). Statistical analysis was performed using the GraphPad Prism 8 software (GraphPad Software Company, San Diego, USA). One-way analysis of variance was used to compare means among groups, and the *t*-test was used to compare means between 2 groups. The percentage and rate were compared using the *χ*^2^ test, and a difference was considered statistically significant at *p* < 0.05.

## 3. Results

### 3.1. AM Could Inhibit the Production of the LPS-Induced IL-6

**Figure 1:**
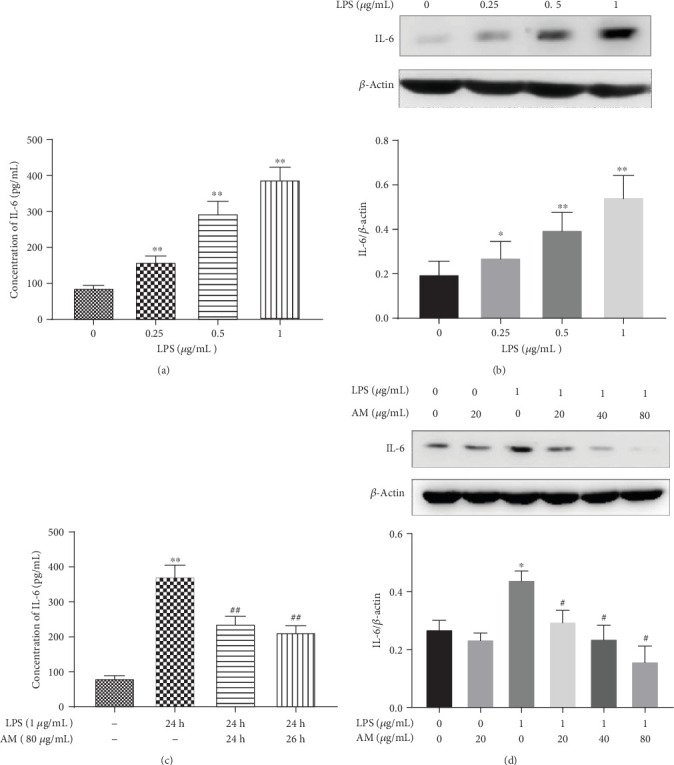
Shift in the production of IL-6 caused by different doses of LPS and AM in ANA-1 cells. (a, c) The concentration of IL-6 in the culture supernatant was detected by ELISA, (b, d) while the protein level of IL-6 in cell extracts was detected by Western blotting, with *β*-actin used as the control. Data represent mean ± SD (*n* = 3). Asterisks (∗) indicate significant differences compared with the control group (^∗^*p* < 0.05, ^∗∗^*p* < 0.01). Number signs (#) indicate significant differences compared with the LPS group (^#^*p* < 0.05, ^##^*p* < 0.01).

ANA-1 cells were stimulated with LPS at various concentrations (0.25, 0.5, and 1 *μ*g/mL) for 24 h. Then, the level of IL-6 in the cell culture supernatant was measured by ELISA, while the protein level of IL-6 in cells was detected by Western blotting. Results showed that LPS increased the levels of both the IL-6 in the cell culture supernatant (Figure 1(a)) and the intracellular IL-6 (Figure 1(b)) in a dose-dependent manner. In order to understand whether the protective effects of AM were manifesting in advance or simultaneously with the stimulation by LPS for 24 h, 2 intervention groups were set up; in one group, AM was added 2 h before LPS, whereas in the other group, AM was added at the same time with LPS. Results showed that AM could downregulate the level of IL-6 in the cell culture supernatant induced by LPS for 24 h (*p* < 0.01), irrespective of whether cells were pretreated with AM 2 h before addition of LPS (in Group AM+LPS) or were cotreated with LPS (in Group LPS+AM) (Figure 1(c)). No significant difference between these 2 groups was observed. In order to observe the reverse effects of AM with LPS, ANA-1 cells were simultaneously exposed to varying concentrations of AM (20, 40, and 80 *μ*g/mL) and 1 *μ*g/mL LPS for 24 h in the same well. Results showed that the LPS-triggered intracellular levels of IL-6 were gradually decreased with increasing concentrations of AM (Figure 1(d)).

### 3.2. LPS Inhibited Autophagy in ANA-1 Cells

**Figure 2:**
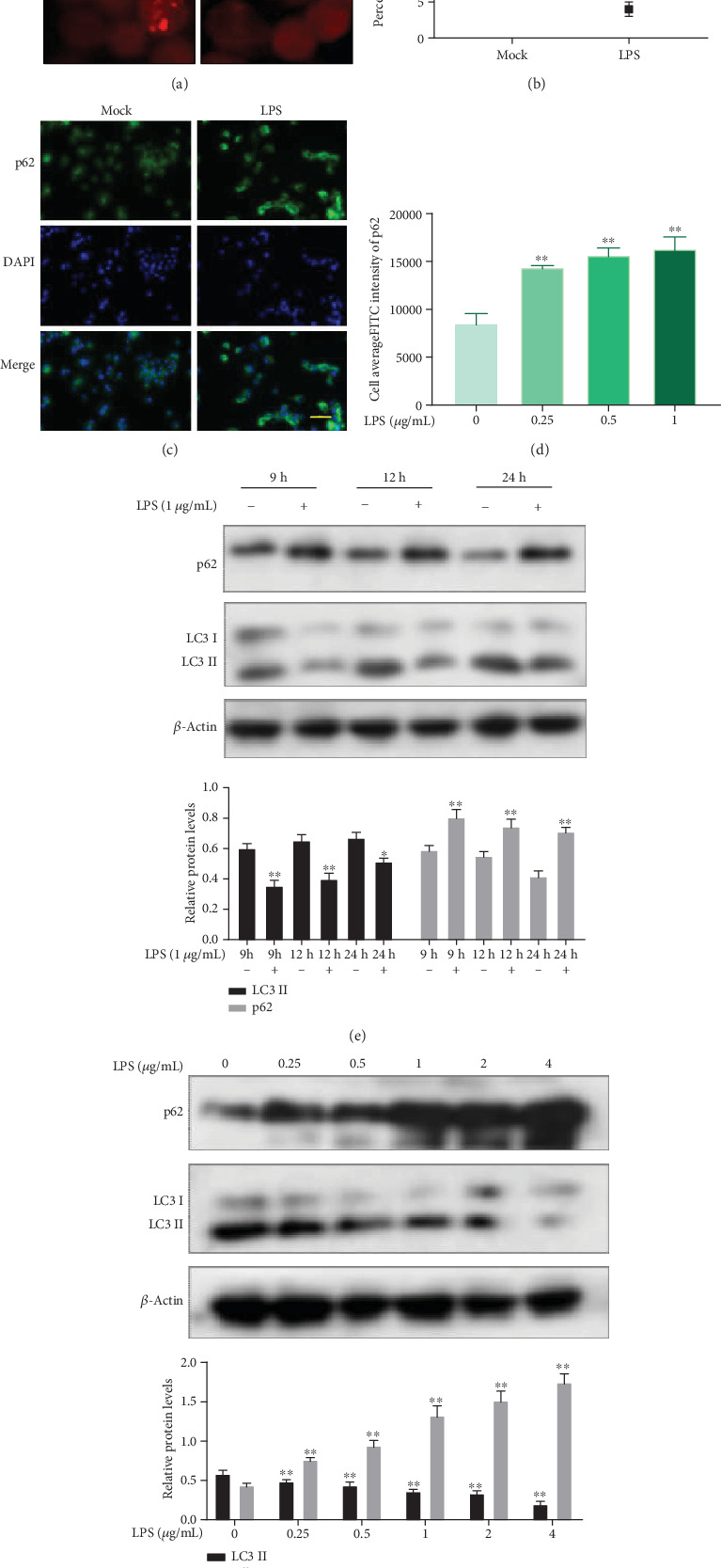
Influence of different doses of LPS on autophagy of ANA-1 cells at varying times of exposure. (a) Red-stained LC3 dots of ANA-1 cells in different groups as shown in the immunofluorescence assay under a fluorescence microscope (Cy3-labeled goat anti-rabbit IgG, LC3 antibody for basement antibody). (b) Percentage of LC3 punctated cells. (c) Green-stained p62 of ANA-1 cells in different groups as shown in the immunofluorescence assay under a fluorescence microscope (FITC-labeled goat anti-rabbit IgG, p62 antibody for basement antibody). Scale bars shown are 50 *μ*m. (d) Analysis with HCS. (e, f) Western blot analysis of changes in the levels of LC3 and p62 proteins in cells. *β*-Actin was used as the control. Data represent mean ± SD (*n* = 3). Asterisks (∗) indicate significant differences compared with the control group (^∗^*p* < 0.05, ^∗∗^*p* < 0.01).

Considering the close link between autophagy and inflammation, we explored the effect of LPS on autophagy. Using immunofluorescence, we detected the punctate intracellular endogenous microtubule-associated protein light chain 3 (LC3) (the specific marker of autophagosomes) and the level of sequestosome 1 (SQSTM1/p62) (a selective substrate of autolysosome). Incubation with 1 *μ*g/mL LPS for 24 h was shown to lead to reduced levels of Cy3-stained LC3 puncta relative to the control group in ANA-1 cells (Figure 2(a)), whereas the levels of FITC-stained p62 were increased (Figure 2(c)). The amount of cells with fluorescent spots of LC3 was counted (at least 50 cells were included for each group), and LPS-treated cells were observed to exhibit a decreased punctate of LC3, compared with the control group (*p* < 0.01) (Figure 2(b)). Then, the fluorescence intensity of FITC-stained p62 was detected with high content imaging systems (HCS), with LPS-treated cells showing increased fluorescence intensity of p62, compared with the control group (*p* < 0.01) (Figure 2(d)). These results demonstrated that LPS inhibited the basic autophagy of ANA-1 cells. The conversion of LC3 I to LC3 II is assumed to be an indicator of autophagy. Therefore, the transformation of LC3 in ANA-1 cells after exposure to LPS was investigated by Western blotting. To detect the impact of LPS on the autophagic flux, the level of p62 was also measured by Western blotting. After LPS treatment at varying times (9, 12, and 24 h), Western blot analysis revealed that LPS inhibited the conversion of LC3 and the degradation of p62 in a time-dependent pattern (Figure 2(e)). It was further shown that at varying concentrations (0.25, 0.5, 1, 2, and 4 *μ*g/mL) of LPS incubated with ANA-1 cells for 24 h, LPS inhibited autophagy in a dose-dependent pattern (Figure 2(f)). These results showed that LPS could lower basic autophagy in macrophagocytes.

### 3.3. Akt/mTOR Pathway Involved in the Inhibition of Autophagy and Production of IL-6 following Treatment with LPS

**Figure 3:**
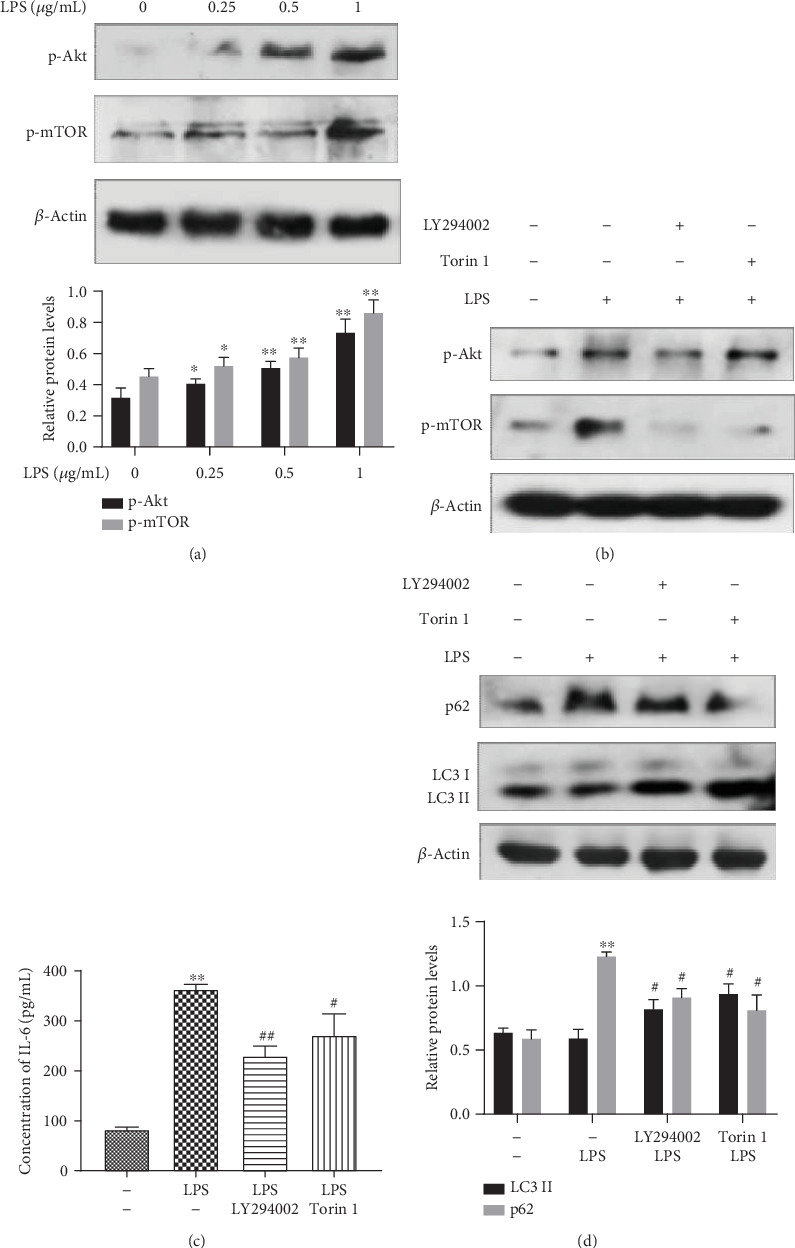
Akt/mTOR mediated the autophagy inhibition caused by LPS in ANA-1 cells. Western blot analysis of the changes in the levels of p-mTOR, p-Akt, LC3, and p62 proteins in ANA-1 cells treated with (a) varying doses of LPS and (b, d) 50 *μ*M LY294002+1 *μ*g/mL LPS and 250 nM Torin 1+1 *μ*g/mL LPS. *β*-Actin was used as the control. (c) Concentration of IL-6 in the culture supernatant detected by ELISA. Data represent mean ± SD (*n* = 3). Asterisks (∗) indicate significant differences compared with the control group (^∗^*p* < 0.05, ^∗∗^*p* < 0.01). Number signs (#) indicate significant differences compared with the LPS group (^#^*p* < 0.05, ^##^*p* < 0.01).

Next, we explored which pathway was involved in the inhibition of autophagy by LPS and whether this pathway could affect the LPS-stimulated production of IL-6. After 24 h of exposure to varying concentrations of LPS (0.25, 0.5, and 1 *μ*g/mL), changes in marker proteins and upstream pathway proteins of autophagy were detected by Western blotting. Results showed that autophagy was inhibited by LPS, and the phosphorylation levels of the mTOR and Akt autophagy pathway proteins were increased in a dose-dependent manner. These effects were more pronounced in the 1 *μ*g/mL LPS group (Figure 3(a)). LY294002 is a potent, cell-permeable inhibitor of Akt acting on the ATP-binding site of the enzyme [61]. Torin 1, a tricyclic benzonaphthyridinone, is also a potent and selective inhibitor of the mTOR kinase [62]. Accordingly, being used as previously reported [63], 50 *μ*M LY294002 was shown to suppress the LPS-stimulated activation of Akt and mTOR. Likewise, being used as previously reported [64], 250 nM Torin 1 suppressed the LPS-stimulated activation of mTOR (Figure 3(b)) and reversed the inhibition of cell autophagy (*p* < 0.05) (Figure 3(d)). As we predicted, inhibition of Akt and mTOR with inhibitors led to the reduced production of the IL-6 level (*p* < 0.01,*p* < 0.05) (Figure 3(c)) in the cell culture medium following stimulation by LPS. These results revealed that LPS could inhibit autophagy by activating the Akt/mTOR pathway.

### 3.4. AM Could Reverse the Inhibitory Effect of LPS on Cell Autophagy

**Figure 4:**
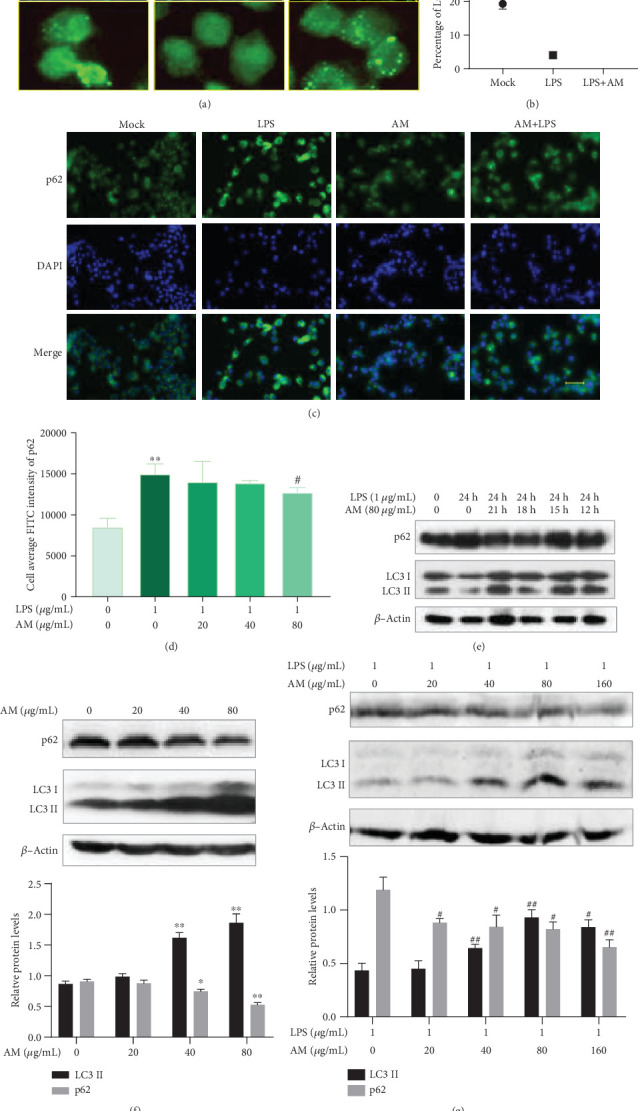
AM can mitigate the inhibitory effect of LPS on autophagy in ANA-1 cells. (a, c) Green-stained LC3 and green-stained p62 of ANA-1 cells in different groups as shown in the immunofluorescence assay (FITC-labeled goat anti-mouse IgG, LC3 antibody and p62 antibody for basement antibody). (b) Percentage of LC3 punctated cells. (e–g) Western blot analysis of the changes in the levels of LC3 and p62 proteins in ANA cells induced by different doses of AM at different coincubation times with or without LPS. *β*-Actin was used as the control. Data represent mean ± SD (*n* = 3). Asterisks (∗) indicate significant differences compared with the control group (^∗^*p* < 0.05, ^∗∗^*p* < 0.01). Number signs (#) indicate significant differences compared with the LPS group (^#^*p* < 0.05, ^##^*p* < 0.01).

Having found that AM could reverse the LPS-induced production of IL-6, we further explored whether it could also reverse the inhibitory effect of LPS on autophagy. The LC3 puncta and the level of p62 were observed by immunofluorescence. It was found that after 24 h incubation with 1 *μ*g/mL LPS, the LC3 puncta were reduced and the level of p62 was increased, whereas addition of 80 *μ*g/mL AM reversed the inhibitory effect of LPS on cell autophagy (Figures 4(a)–4(c)). Next, the fluorescence intensity of FITC-stained p62 was detected with HCS. Respectively, LPS-treated cells showed enhanced fluorescence intensity of p62 compared with the control group (*p* < 0.01), whereas treatment with 80 *μ*g/mL AM attenuated the fluorescence intensity of p62 compared with the LPS group (*p* < 0.05) (Figure 4(d)). To further confirm the reverse effect of AM to the LPS-induced inhibition, we observed the changes of autophagy in LPS-treated cells with varying times of treatment with AM. First, we added 1 *μ*g/mL LPS to each group, and then, 80 *μ*g/mL AM was, respectively, added to the cell culture medium of different groups at 3, 6, 9, and 12 h after the addition of LPS. Cells were incubated for 24 h since the addition of LPS and then were collected for Western blot analysis. Results showed that earlier treatment with AM produced the stronger reverse effect of AM on the LPS-induced inhibition of autophagy (Figure 4(e)). Next, we tested the antagonistic effect of varying doses of AM on LPS-induced inhibition of autophagy. Cells were stimulated with or without 1 *μ*g/mL LPS and varying concentrations of AM for 24 h. We found that increasing concentrations of AM resulted in increased autophagy with or without LPS stimulation (Figures 4(f) and 4(g)). These results suggested that AM could induce autophagy, as well as reverse the inhibitory effect of LPS on cell autophagy.

### 3.5. AM Inhibited mTOR through Activating AMPK, rather than Inhibiting Akt

**Figure 5:**
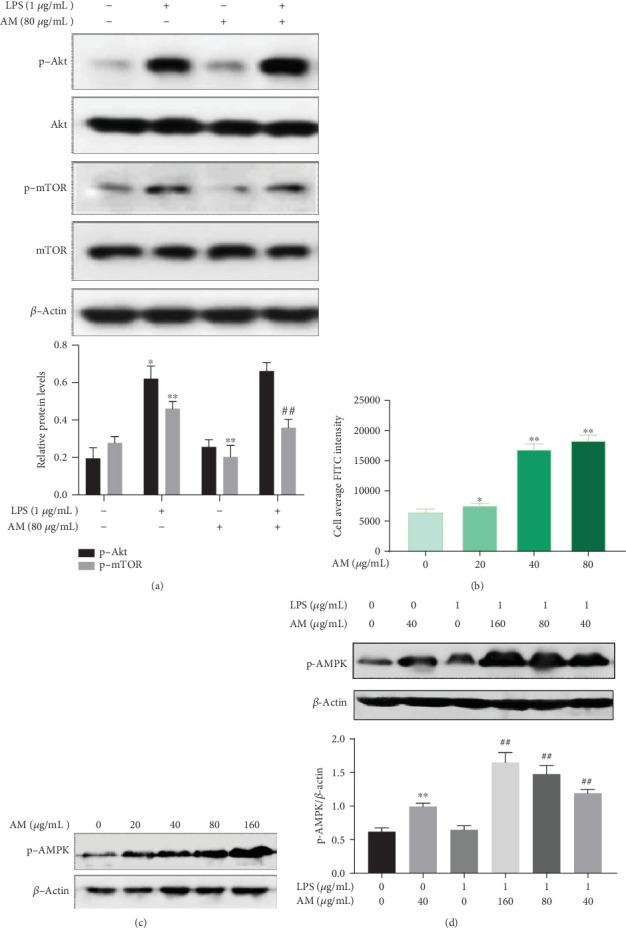
AM inhibited mTOR by activating AMPK in ANA-1 cells. (a) Western blot analysis of the effects of AM on p-mTOR and p-Akt proteins in ANA-1 cells. (b–d) The effects of AM on the phosphorylation of AMPK at varying doses in ANA-1 cells. Data represent mean ± SD (*n* = 3). Asterisks (∗) indicate significant differences compared with the control group (^∗^*p* < 0.05, ^∗∗^*p* < 0.01). Number signs (#) indicate significant differences compared with the the LPS group (^#^*p* < 0.05, ^##^*p* < 0.01).

Based on previous research, we hypothesized that the antagonistic effect of AM on LPS might be mediated by the opposite regulation of the Akt/mTOR pathway. We performed Western blot analysis to further test and replicate the effect of LPS on Akt and mTOR. Interestingly, we found that AM did not lead to a decrease in the level of phosphorylated Akt but reduced the phosphorylation of mTOR with or without LPS stimulation (*p* < 0.01) (Figure 5(a)). Thus, we concluded that the inhibitory effect of AM on mTOR was not performed through Akt, and it suggested that AM might play a regulatory role on mTOR through other pathways. Then, we sought to identify these signaling pathways by performing immunofluorescence assays and HCS and found that AM could induce the phosphorylation of AMPK (Figure 5(b)), which is one of the upstream targets of mTOR. After treatment of ANA-1 cells with AM at various concentrations (20, 40, and 80 *μ*g/mL) for 24 h, we noted that the phosphorylation level of AMPK was increased in a dose-dependent manner (Figure 5(c)). The following result was not surprising: administration of AM together with LPS was shown to enhance the level of p-AMPK in a dose-dependent manner, compared with the LPS group (*p* < 0.01) (Figure 5(d)). These results suggested that AM could inhibit mTOR by activating AMPK, thereby inducing autophagy and resisting the LPS-induced inhibition of cell autophagy.

### 3.6. Activation of AMPK Could Inhibit the LPS-Induced Production of IL-6

**Figure 6:**
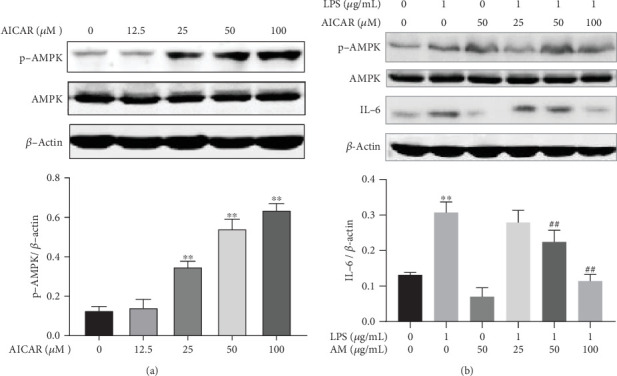
The AMPK agonist reduced the level of IL-6 in ANA-1 cells. (a) Western blot analysis detecting the activating effect of AICAR on AMPK. *β*-Actin was used as the control. (b) Western blot analysis of the effect of varying doses of AICAR on the level of IL-6 in ANA-1 cells. *β*-Actin was used as the control. Data represent mean ± SD (*n* = 3). Asterisks (∗) indicate significant differences compared with the control group (^∗^*p* < 0.05, ^∗∗^*p* < 0.01). Number signs (#) indicate significant differences compared with the LPS group (^#^*p* < 0.05, ^##^*p* < 0.01).

The above experimental results showed that AM could antagonize the inhibitory effect of LPS on autophagy and the production of IL-6 by activating AMPK. We therefore explored whether AMPK agonists could produce similar effects with those shown by AM. Acadesine (AICAR), an AMPK activator [65], was administered to ANA-1 cells at concentrations of 12.5, 25, 50, and 100 *μ*M for 24 h. Results of Western blot analysis showed that 25, 50, and 100 *μ*M AICAR increased the phosphorylation level of AMPK in ANA-1 cells, compared with the control group (*p* < 0.01) (Figure 6(a)). ANA-1 cells were then incubated with AICAR at concentrations of 25, 50, or 100 *μ*M and 1 *μ*g/mL LPS for 24 h. Compared with LPS intervention alone, 50 and 100 *μ*M AICAR were shown to significantly enhance the phosphorylation level of AMPK (Figure 6(b)). Next, we observed the effect of AICAR on the LPS-induced production of IL-6. Western blot analysis showed that the LPS-induced production of IL-6 was decreased by AICAR at concentrations of 50 and 100 *μ*M (*p* < 0.01) (Figure 6(b)). These results confirmed that the AMPK activator could play a similar role with AM in inhibition of IL-6 production.

### 3.7. The Effect of AM on Activation of Autophagy and Inhibition of IL-6 Could Be Blocked by Knocking Down AMPK

**Figure 7:**
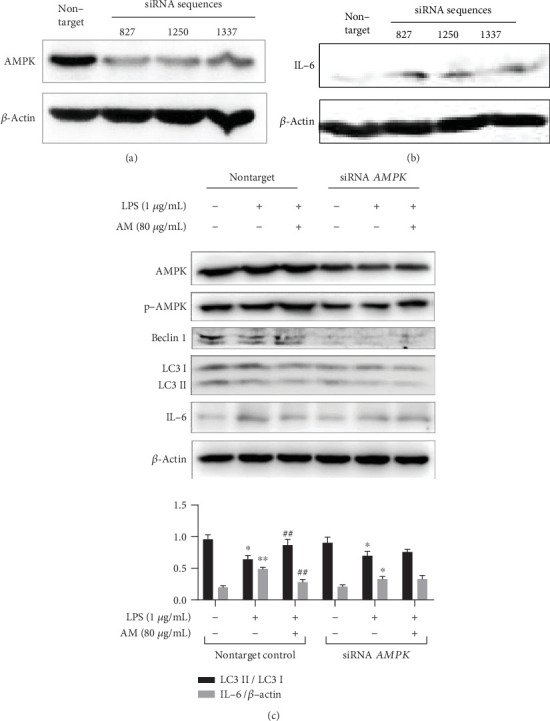
siRNA *AMPK* reversed the AM-induced autophagy and inhibition of IL-6 production in ANA-1 cells. (a) AMPK siRNA reduced the level of p-AMPK, (b) whereas increased the level of IL-6, in the cell. (c) Western blot analysis of the effects of AM on AMPK, Beclin 1, LC3, p-AMPK, and IL-6 proteins in ANA-1 cells before and after knocking down AMPK. *β*-Actin was used as the control. Data represent mean ± SD (*n* = 3). Asterisks (∗) indicate significant differences compared with the control group (^∗^*p* < 0.05, ^∗∗^*p* < 0.01). Number signs (#) indicate significant differences compared with the LPS group (^#^*p* < 0.05, ^##^*p* < 0.01).

In order to further demonstrate that the inhibitory effect of AM against LPS was accomplished through the activation of AMPK, we compared the effect of AM on autophagy and IL-6 between an AMPK knocked-down group and a nontarget siRNA control group. Three specific siRNA sequences (starting sites: 827, 1250, and 1337 bp, respectively) targeted at the *AMPK* gene were synthesized. After transfecting ANA-1 cells with these sequences, we observed that AMPK was downregulated (Figure 7(a)) and this was accompanied by an increase in the level of IL-6 (Figure 7(b)). The highest efficiency was exhibited by the interference sequence with the starting site at 1250 bp, and thus, this fragment was used in subsequent interference experiments. In the nontarget siRNA control group, LPS reduced the level of Beclin 1, decreased the conversion of LC3, and increased the level of IL-6 in cells (*p* < 0.01). In the case where the levels of AMPK and p-AMPK were knocked down by specific siRNAs, administration of AM could not reverse the effects of LPS. These findings showed that the activation of AMPK might play a key role in AM-induced autophagy and inhibition of IL-6 production.

### 3.8. Activation of Autophagy Could Inhibit the Production of IL-6

**Figure 8:**
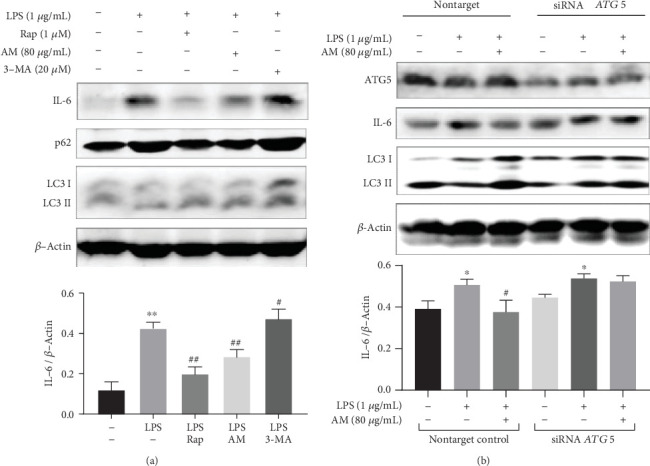
Activation of autophagy can reduce the expression level of IL-6. (a) Western blot analysis detecting the effects of Rap and 3-MA on the level of IL-6 in ANA-1 cells. *β*-Actin was used as the control. (b) Western blot analysis detecting the effects of AM and LPS on ATG5, LC3, and IL-6 proteins in ANA-1 cells before and after knocking down *ATG5*. *β*-Actin was used as the control. Data represent mean ± SD (*n* = 3). Asterisks (∗) indicate significant differences compared with the control group (^∗^*p* < 0.05, ^∗∗^*p* < 0.01). Number signs (#) indicate significant differences compared with the LPS group (^#^*p* < 0.05, ^##^*p* < 0.01).

The previous results showed that all similarly induced (LPS or AM) changes in autophagy and expression of IL-6 pass through the same signaling pathway. This drove us to further explore the relationship between autophagy and production of IL-6. To clarify the effect of autophagy on the level of expression of IL-6, we used rapamycin (Rap), an autophagy inducer, and 3-methyladenine (3-MA), an autophagy inhibitor, to influence the level of autophagy and observed the changes of IL-6 in LPS-induced macrophages. Results showed that Rap (1 *μ*M) was performed as previously reported [64] and led to activation of autophagy and decreased levels of LPS-induced IL-6, compared with the LPS group (*p* < 0.01). On the other hand, the 3-MA (20 *μ*M) autophagy inhibitor was shown to cause an increase in the expression of IL-6, compared with the LPS group (*p* < 0.05) (Figure 8(a)). Next, we used siRNA to interfere with the expression of the autophagy-related gene (*ATG5*) to inhibit initiation of autophagy and observe the changes in the levels of IL-6. Results showed that knockdown of *ATG5* resulted in inhibition of autophagy and increased expression of IL-6 compared with the nontarget group. Moreover, it was shown that AM could not reduce the LPS-induced production of IL-6 due to the blocking of the autophagy signaling pathway (Figure 8(b)). These results indicated that AM might play an inhibiting role in the expression of IL-6 due to activation of autophagy.

## 4. Discussion

In this study, we showed that the production of IL-6 was elevated after incubation of ANA-1 cells with LPS. However, we also observed that this response was reversed by treatment with AM. In our previous study, we used LPS to establish an inflammation model in mice, which were then treated with AM injection to detoxify LPS. Our results showed that the LPS-exposed mice displayed a significant weight loss, a temperature increase, splenomegaly, hemorrhage, pulmonary hyperemia, and an increase in serum levels of IL-6. Respectively, AM injection was demonstrated to reduce this LPS-induced inflammatory response and decreased the levels of IL-6 in the serum of mice (Supplementary Figure 1). IL-6 is known to be the earliest inflammatory marker and has been reported to also function as an indicator of therapeutic effects and prognosis marker of inflammatory diseases [66]. Subsequently, we confirmed the suppressive effect of AM on the LPS-induced production of IL-6 in inflammatory macrophages.

After confirming the anti-inflammatory effect of AM, we aimed to explore its anti-inflammatory mechanism. To this end, we first sought to identify other regulatory effects of LPS on macrophages. Deretic et al. [67] discussed in a review that autophagy is a potent anti-inflammatory process through its regulatory interactions with signaling pathways. On that note, we attempted to explore whether there was a relationship between inflammation and autophagy in our experimental model. Although some studies suggested that LPS could induce autophagy in bone marrow-derived macrophages [68], our immunofluorescence and Western blotting results demonstrated that LPS inhibited autophagy in macrophages, consistent with the study by Hu et al. [69]. Recently, more studies have affirmed that LPS can inhibit autophagy in hepatocytes [70, 71], RAW 264.7 macrophages [72, 73], and mouse articular chondrocytes [74]. Different effects on autophagy might result from different interventions applied to different types of cells. We speculated that activation of autophagy under the intervention of LPS might be a self-protective stress mechanism of cells, and once LPS dominates on cells, its inhibitory effect on autophagy would become apparent. This conflict should be explored in further studies.

A major player in autophagy is known to be the mTOR protein, which is a signaling control point downstream of the PI3K/Akt, MAPK/ERK 1/2, AMPK, and P53/genotoxic stress pathways. Importantly, mTOR has been acknowledged to act in inhibiting autophagy under the activation of PI3K/Akt or MAPK/ERK and the inhibition of the AMPK and P53/genotoxic stress pathways [75]. Respectively, we found that LPS inhibited autophagy by activating Akt/mTOR signaling. Results showed that administration of inhibitors of mTOR and its upstream protein kinase led to the same effects on autophagy and secretion of IL-6, as those observed for AM. We speculated that AM might resist the effect of LPS by inhibiting Akt/mTOR; however, our experimental results showed the unexpected effect that AM lowered the levels of p-mTOR without inhibiting the Akt kinase.

Next, we found that among the upstream activation pathways of autophagy, AM could activate AMPK and induce autophagy. AMPK is known to be a key metabolic regulator in cells. In the absence of energy, activated AMPK has been reported to promote catabolism by phosphorylating downstream proteins, thereby preserving ATP levels in cells. Activated AMPK has also been shown to trigger autophagy by inhibiting mTOR [76, 77]. The inhibitory effect of AMPK on inflammation has been demonstrated by several studies [78–80], mainly focused on its regulation of Nrf2 [81], SIRT1 [82, 83], and PGC-1 [84, 85] and effect on the functions of various inflammation-related proteins, such as NF-*κ*B and AP-1. Therefore, AMPK could be used as a potential target for the treatment of inflammation and other related diseases. We further speculated that the activation of AMPK was involved in the inhibitory effect of AM on the production of IL-6. Our results demonstrated that AICAR, an AMPK activator, yielded similar effects in resisting LPS as those seen in the case of treatment with AM. Subsequently, we used siRNA to knock down the expression of AMPK in ANA-1 cells and found that the effects of AM on cell autophagy and production of IL-6 were reduced by the *AMPK*-targeted siRNA, further demonstrating that AM could exert its anti-inflammatory effect due to the activation of AMPK. Guma et al. [86] found that activation of AMPK suppressed the expression of IL-6 in serum and arthritic joints of mice with inflammatory arthritis. Despite the different models of inflammation, results from this and other studies [87, 88] supported our conclusion and suggested that targeted activation of AMPK has a potential to be an effective therapeutic strategy for IL-6 dependent inflammatory diseases.

AMPK is known to be one of the major mediators of autophagy. Our results showed that AM induced autophagy by activating AMPK and reduced the LPS-induced production of IL-6, suggesting that there should be some interaction between autophagy and anti-inflammatory effects. We attempted to further clarify whether activation of AMPK played an anti-inflammatory role through the upregulation of autophagy and got a positive answer. Other studies [89–92] have shown that activating autophagy through different pathways could exert anti-inflammatory effects, in agreement with our results. Autophagy is an important regulatory mechanism for maintaining homeostasis in the body. Many experimental studies have also shown that physiological activation of autophagy could improve metabolism, immune function, and anticancer and anti-inflammatory functions of the body [71, 93]. Melendez et al. [94] demonstrated for the first time that the *bec1* (called *ATG6* in yeast and Beclin 1 or *BECN1* in mammals) autophagy gene was necessary in the longevity type of the daf-2 mutant in *C*. *elegans*. Recently, Fernández et al. [95] found that activation of autophagy resulted in the increased lifespan of mammals. We speculated that other roles of AM might be closely related to autophagy regulatory mechanisms in cells. Based on these findings, other Chinese medicine and natural foods able to induce autophagy could be exploited toward the development of anti-inflammatory drugs and functional health foods.

In future studies, we will focus on identifying the major active components in AM exerting the anti-inflammatory effects and further verify their mechanisms of action in both the in vitro and in vivo inflammation models, as well as search for possible anti-inflammatory targets.

## 5. Conclusions

**Figure 9:**
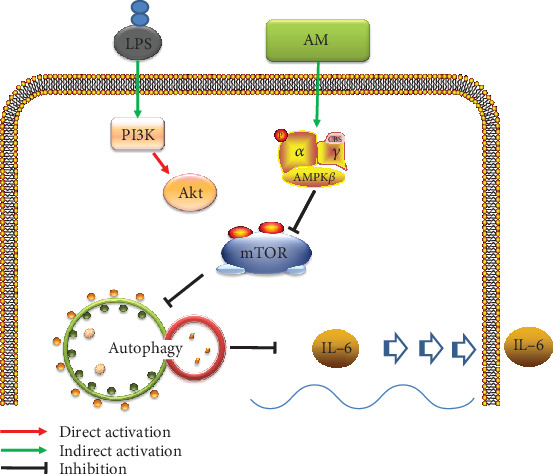
Proposed mechanisms of AM on the expression of IL-6 in LPS-stimulated macrophages. LPS inhibits autophagy by activating the PI3K/Akt/mTOR pathway and also upregulates the expression of IL-6. The AM inhibits mTOR, promoting autophagy, and inhibits the expression of IL-6 by increasing the phosphorylation of AMPK. The activation of autophagy leads to the inhibition of the expression of IL-6. Conclusively, AM inhibits the expression of IL-6 and promotes autophagy via modulating the AMPK-mTOR signaling pathways.

In this study, we demonstrated that LPS inhibited autophagy in the ANA-1 murine macrophages and induced the production of the IL-6 inflammatory cytokine by activating the Akt/mTOR pathway. Interestingly, AM induced cell autophagy and reduced the production of IL-6 in ANA-1 cells with or without stimulation by LPS. However, AM induced autophagy not through the direct inhibition of Akt/mTOR but instead by triggering the AMPK-mediated inhibition of the mTOR pathway. Hence, AM and LPS exerted opposite effects on autophagy and the production of IL-6 through different upstream signaling pathways of mTOR. In addition, the AM-induced autophagy played a role in this anti-inflammatory effect. All these interactions are given in a proposed mechanism illustrated in Figure 9.

## Data Availability

The data used to support the findings of this study are available from the corresponding author upon request.

## Supplementary Materials

Supplementary Materials Supplementary Figure 1: AM injection can inhibit the inflammation response induced by LPS in mice. (a) The weight of mice after LPS was measured at different time points. (b) The levels of serum IL-6 in mice from the LPS treatment and LPS with AM injection treatment groups, analyzed at different time points by enzyme-linked immunosorbent assay (ELISA). (c, d) The spleen and lung of mice were isolated, and the pathological effect was observed. Supplementary Video 1: after 24 hours of lipopolysaccharide injection, the action activity of mice was compared with the control group. The mice in the saline control group had normal diet and active exercise, while the mice in the lipopolysaccharide treatment group had obvious signs of debility, tremor, and loss of appetite.
