# Supplementary material for: Astragalus membranaceus Injection Suppresses Production of Interleukin-6 by Activating Autophagy through the AMPK-mTOR Pathway in Lipopolysaccharide-Stimulated Macrophages
Source: Oxid Med Cell Longev. 2020 Jul 4;2020:1364147. doi: 10.1155/2020/1364147 (PMC7364262; doi:10.1155/2020/1364147)
Supplement: Supplementary Materials — Supplementary Figure 1: AM injection can inhibit the inflammation response induced by LPS in mice. (a) The weight of mice after LPS was measured at different time points. (b) The levels of serum IL-6 in mice from the LPS treatment and LPS with AM injection treatment groups, analyzed at different time points by enzyme-linked immunosorbent assay (ELISA). (c, d) The spleen and lung of mice were isolated, and the pathological effect was observed. Supplementary Video 1: after 24 hours of lipopolysaccharide injection, the action activity of mice was compared with the control group. The mice in the saline control group had normal diet and active exercise, while the mice in the lipopolysaccharide treatment group had obvious signs of debility, tremor, and loss of appetite. [file 1364147.f1.zip › Supplementary Figure 1 and video 1.docx]

Supplementary materials

Supplementary Figure 1 (Figure S 1) Legends

1. Animals

Male C57BL/6 mice weighing 20-22g at the age of 5 weeks were used as experimental animals. They were kept under controlled temperature (21–23°C) and humidity (45–65%). The room was lit from 8:00 am to 8:00 pm and during the behavioral test. Food and water were available ad libitum. The study was approved by the Committee for Animal Experiments at the Fenyang College of Shanxi Medical University (Fenyang, China). Mice were injected into the tail vein with 2mg/mL LPS, 0.1mL/mouse. 12 or24 hours after injection, AM injection was injected into the tail vein of mice for treatment.

2.Results

In order to observe the pathogenicity of lipopolysaccharide (LPS) and the protective effect of Astragalus membranaceus injection, we carried out animal experiments in mice. Firstly, mice were injected into the tail vein with 2mg/mL LPS, 0.1mL/mouse. The mice were weighed at 6, 12, 24, 36, 48 and 60 hours after LPS injection, and the control group was injected with sterile normal saline (NS). As shown (Figure S 1 (a)), the body weight of the control group did not change significantly with the increase with time, while the body weight of the LPS group decreased from about 6 hours to the lowest at about 36 hours, and then increased and return to normal weight after 60 hours approximately. Next, we observed the effect of AM injection on serum IL-6 of mice, and found that after 12 hours of LPS injection, the serum concentration of IL-6 in mice increased significantly. The mice were treated with AM injection (0.1 mL / mouse) and normal saline injection (0.1 mL / mouse) as control group. After 12 hours of treatment, the serum level of IL-6 in both two groups was decreased, but it was still higher than that before injection. Compared with the NS control group, the serum level of IL-6 in the AM injection group was decreased significantly (Figure S 1 (b)). In addition, we observed the effects of LPS and AM injection on the spleen and lung in mice.

Group A was the healthy control group, group B was the LPS inflammation model group, and group C was the AM injection treatment group. After 24h from LPS injecting, AM injection was used for treatment. Group C #3.1, C #.2, C #.3 (# stands for the number 1,2, or 3) are respected low, medium, high three treatment dose groups (three doses: 0.05mL, 0.1mL and 0.2mL). Group C 1.#, C 2.#, C 3.# (# stands for the number 1,2, or 3) are respected 1d, 2d, 3d treatment with a certain dose. We found that the LPS-induced inflammatory mice had enlarged spleens, dark lesions, and hyperemia of the lungs compared to the healthy mice. After treated with AM injection, inflammatory lesions in the spleen and lungs of mice were reduced (Figure S 1 (c)), and improved gradually with the increase of treatment frequency and dose (Figure S 1 (d)).


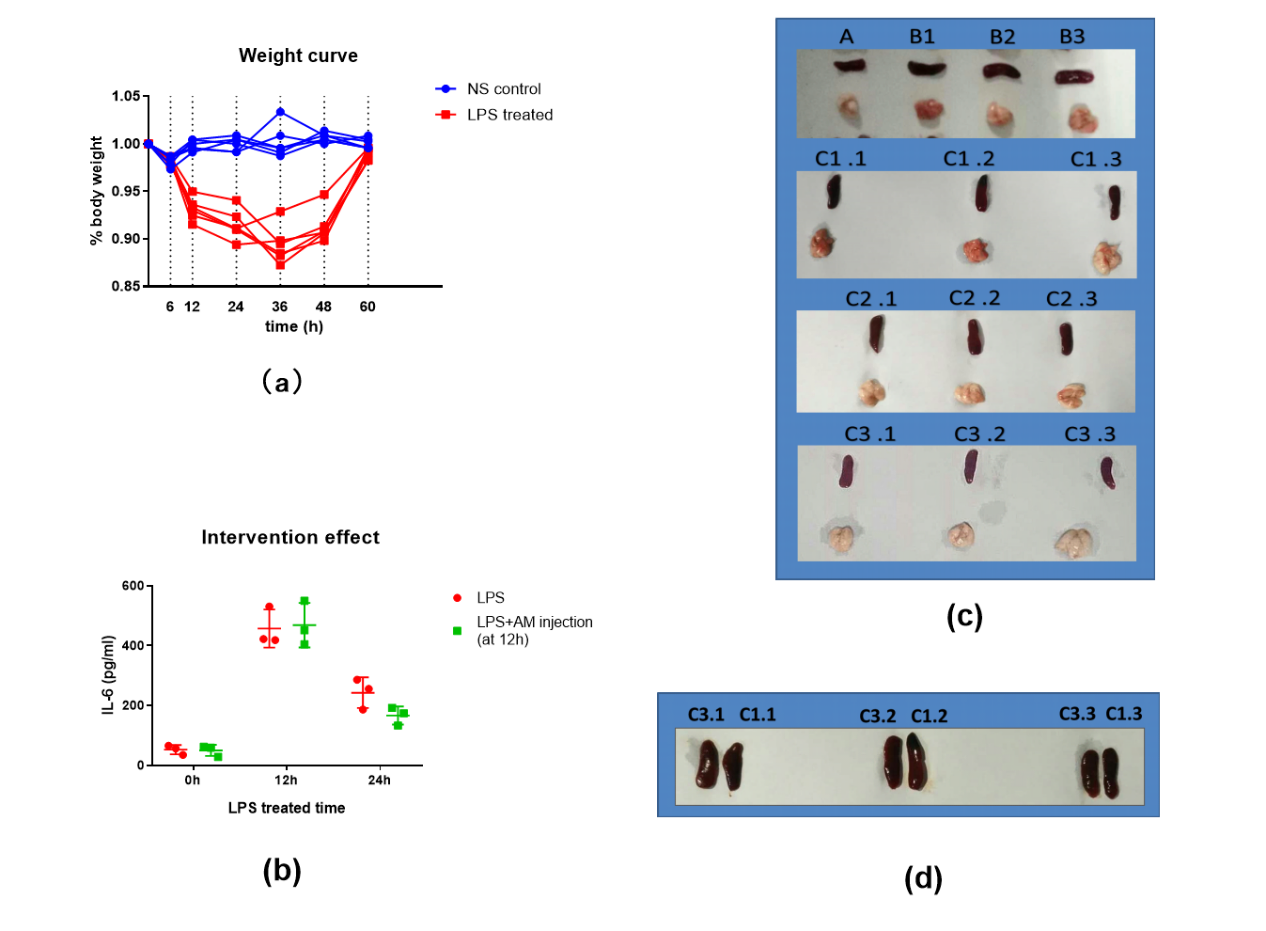


Supplementary Figure 1 AM injection can inhibit the inflammation response induced by LPS in mice. (a) The weight of mice after LPS was measured at different time points. (b) The levels of serum IL-6 in mice from LPS treatment and LPS with AM injection treatment groups, analyzed at different time points by enzyme-linked immunosorbent assay (ELISA). (c and d) The spleen and lung of mice were isolated and the pathological effect was observed.

Supplementary Video 1

After 24 hours of lipopolysaccharide injection, the action activity of mice was compared with control group. The mice in the saline control group had normal diet and active exercise, while the mice in the lipopolysaccharide treatment group had obvious signs of debility, tremor and loss of appetite.
